# Clustering and switching in verbal fluency: a comparison between control and individuals with brain damage

**DOI:** 10.1590/2317-1782/20212020365

**Published:** 2021-11-22

**Authors:** Karina Carlesso Pagliarin, Eduarda Giovelli Fernandes, Maryndia Diehl Muller, Caroline Rodrigues Portalete, Rochele Paz Fonseca, Raira Fernanda Altmann

**Affiliations:** 1 Programa de Pós-Graduação em Distúrbios da Comunicação Humana, Universidade Federal de Santa Maria – UFSM, Santa Maria (RS), Brasil.; 2 Programa de Pós-Graduação em Psicologia, Pontifícia Universidade Católica do Rio Grande do Sul – PUCRS, Porto Alegre (RS), Brasil.

**Keywords:** Language Tests, Stroke, Evaluation, Adults, Verbal Fluency, Testes de Linguagem, Acidente Vascular Cerebral, Avaliação, Adultos, Fluência Verbal

## Abstract

**Purpose:**

The aim of this study is to analyze and compare the performance and strategies used by control subjects and patients with unilateral brain damage on phonemic and semantic Verbal Fluency tasks.

**Methods:**

The sample consisted of 104 participants divided into four groups (26 with left hemisphere damage and aphasia- LHDa, 28 with left hemisphere damage and no aphasia- LHDna, 25 with right hemisphere damage- RHD and 25 neurologically healthy control subjects). All participants were administered the phonemic (“M” letter-based) and semantic (animals) verbal fluency tasks from the Montreal-Toulouse Language Assessment Battery (MTL-BR).

**Results:**

Patients in the LHDa group showed the worst performance (fewer words produced, fewer clusters and switches) in both types of fluency task. RHD group showed fewer switching productions when compared with controls and LHDna had fewer words productions than controls in the first 30 seconds block.

**Conclusion:**

Our findings suggest that the LHDa group obtained lower scores in most measures of SVF and PVF when compared to the other groups.

## RESUMO

**Objetivo:**

analisar e comparar o desempenho e as estratégias utilizadas por indivíduos controle e pacientes com lesão cerebral unilateral em tarefas de Fluência Verbal fonêmica e semântica.

**Método:**

A amostra foi composta por 104 participantes, divididos em quatro grupos (26 com lesão no hemisfério esquerdo e afasia - LHEa, 28 com lesão no hemisfério esquerdo e sem afasia - LHEna, 25 com lesão no hemisfério direito - LHD e 25 controles neurologicamente saudáveis). Todos os participantes realizaram tarefas de fluência verbal fonêmica – FVF (com letra “M”) e semântica- FVS (animais) da Bateria Montreal-Toulouse de Avaliação da Linguagem (MTL-BR).

**Resultados:**

Pacientes do grupo LHEa apresentaram pior desempenho (menos palavras produzidas, menos clusters e switching) nos dois tipos de tarefas de fluência. O grupo LHD mostrou menos produções de switching quando comparado aos controles. O grupo LHEna teve menos produções de palavras que os controles no primeiro bloco de 30 segundos.

**Conclusão:**

Nossos achados concluem que o grupo LHEa obteve escores mais baixos na maioria das medidas de FVS e FVF quando comparado aos demais grupos.

## INTRODUCTION

Unilateral brain damage is associated with several types of communicative-linguistic impairment. Right hemisphere damage (RHD) may affect pragmatics, prosody, semantics and even complex discursive skills such as metaphor comprehension^(1,2)^. Left-hemisphere damage (LHD), on the other hand, is associated with impairments in phonology, morphology, syntax and semantics, all of which are typical symptoms of classic aphasia. These alterations result in a series of observable linguistic behaviors, including anomia, speech suppression, paraphasia, agrammatism, and neologisms^(3,4)^.

Some of the most common tools in the assessment of neurological impairment are verbal fluency (VF) tasks, which allow the clinician to evaluate a number of cognitive domains, including language, semantic memory and executive functioning, in a few minutes or less. Given their speed and ease of administration, as well as their reliability in the assessment of cognitive abilities, VF tasks are widely used in both inpatient and outpatient settings^(5,6)^.

Fluency tasks can be either verbal or non-verbal (drawing). Verbal forms of the task usually involve the production of a series of words according to a predetermined criterion, as in the case of semantic (SVF) and phonemic (PVF) verbal fluency. In SVF, the subject must elicit words within a semantic category, such as fruits, animals or items of clothing. In PVF, the participant is asked to elicit as many words as possible starting with a given letter over the course of a pre-established time period, usually of 1 minute^(7)^. The SVF tasks are used to verify the functioning of the temporal lobe, while PVF tasks used to verify the functioning of the frontal lobe^(8)^.

VF tasks provide quantitative scores, such as the total number of correct words elicited by the individual, excluding repetitions and errors as well as qualitative performance indicators, which relate to the production strategies used by the subject over the course of the task, and are commonly known as clustering and switching measures. Clustering is an executive-linguistic subprocess, in which the cluster is a group of two or more consecutively generated words belonging to the same semantic or phonetic category. Some examples include types of animals from a particular location (farm, zoo, jungle). It is still possible to form clusters of words that have the same rhyme or that begin with the same letter. On the other hand, switching refers to the ability to shift from one category to another, number of times in which there is a change from one type of cluster to another^(6,8-11)^.

For proper VF task performance, both clustering and switching components are required^(12)^. Clustering is associated with the cerebral functioning of the temporal lobe that is related to semantic verbal memory and word storage. The switching component is associated with the frontal lobe that is related to cognitive flexibility and word processes search^(8)^.

Regarding the number of switches and clusters, both are related to each other, because by identifying the number of clusters, you can get the number of switches (corresponds to the number of clusters minus one)^(12)^. The quantitative interpretation although very classic, is not enough for a deeper analysis of the cognitive-linguistic strategies used by the patients. Furthermore, quantitative analysis may not differentiate patients in the total number of correct words. Thus, there may often be no differences in the total correct word score, but in contrast there may be poorer strategies following a neurological condition such as stroke in the left hemisphere^(13)^ or traumatic brain injury (TBI)^(14)^.

The qualitative analysis of VF tasks allows the examiner to investigate several cognitive skills, including executive functioning, initiation, lexical search and retrieval, organization of search strategies, inhibition, working memory, and cognitive flexibility^(5,10)^. In addition to contributing to the detection of executive dysfunction in neurological conditions, the qualitative analysis of VF tasks may help develop treatment strategies for patients in need of neuropsychological rehabilitation^(12)^.

It is well established that aphasic patients produce fewer words than healthy controls in this kind of task, but there are few researches with regard to the qualitative nature of the performance^(10)^.

It is important to highlight that clustering and switching scores can also reveal which aspects of language processing are impaired or unaffected after brain damage, contributing to a final diagnosis. Additionally, measures of performance over time allow for an assessment of faster, automatic processes, as well as those which are slower and require additional cognitive effort, resulting in controlled processes which place greater demands on attentional resources^(15)^. In light of these observations, the aim of this study was to analyze and compare the performance and strategies used by control subjects and patients with unilateral brain damage on phonemic and semantic VF tasks.

## METHODS

### Participants

The participants were recruited from hospitals settings and evaluated at the school clinic of a higher education institution or at the hospital's neurology outpatient clinic. All participants were assessed individually in a single session.

The sample consisted of 104 adults, were divided into four groups, consisting of the following: 26 participants with LHD and aphasia (LHDa group), 25 with RHD (RHD group), 28 with LHD and no aphasia (LHDna) and 25 neurologically healthy adults (Control group). Groups were matched for age (sample M=58.50; SD=12.39) and education (sample M=9.98; DP=5.76) (Table 1).

**Table 1:** Sociodemographic characteristics of participant groups

	LHDa (*n* = 26)		RHD (*n* = 25)		LHDna (*n* = 28)		C (*n* = 25)		*F*(3,100)	*X^2^* (3,6)	*p*
	*M*	*SD*	*M*	*SD*	*M*	*SD*	*M*	*SD*			
Age (years)	60.35	10.57	59.28	14.23	57.68	12.48	56.60	12.51	.454		.715
Education (years)	9.77	5.72	9.12	5.01	10.43	5.65	10.08	6.10	.254		.858
Sex											
Female (%)	8 (30.80%)		12 (48.00%)		13 (46.40%)		14 (56.00%)			3.459	.326
Male (%)	18 (69.20%)		13 (52.00%)		15 (53.60%)		11 (44.00%)				

**Caption:** LHDa = Left hemisphere damage, aphasia; RHD = Right hemisphere damage; LHDna = Left hemisphere damage, no aphasia; C = Control group; X*^2^* = Chi-square; M= Mean; SD=Standard deviation; F = F-statistic.

Table 1 shows that participants were paired considering age, education and sex.

This research was carried out at a higher education institution. The following inclusion criteria were applied to the sample: being first-language Portuguese speakers; at least 19 years of age; right-handedness according to the Edinburgh Inventory^(16)^; no uncorrected sensory (visual and/or hearing) impairments; and no previous or current psychoactive substance use^(17)^.

In addition to these criteria, participants in the control group were screened for neurological disorders. Clinical participants were required to have been diagnosed with a unilateral ischemic stroke at least six months prior to the study. The specific location of the lesion was confirmed by neuroimaging tests. The subjects with aphasia were previously diagnosed by the Montreal-Toulouse Language Assessment Battery (MTL-BR)^(4)^. None of the patients had previously undergone lexical-semantic treatment. The location of cerebral lesions as per magnetic resonance imaging (MRI) or computed tomography, as well as the classification of patients with aphasia, are described in Chart 1.

**Chart 1:** Lesion location according to magnetic resonance imaging or computed tomography, and aphasia classification

Lesion Location	Type of aphasia/lesion	Number of patients
Left MCA; frontal (left opercular); left fronto-temporal-parietal region; left insula	Broca’s aphasia	7
Left supplementary motor area	Transcortical motor aphasia	1
Left temporal posterior cerebral artery	Transcortical sensory aphasia	1
Left MCA; left temporo-parietal region	Wernicke	2
Left temporo-occipital region; left semioval centers; left MCA.	Conduction aphasia	4
Left parietal region; left anterior temporal region	Anomic aphasia	4
Left MCA; left temporo-parietal region; parieto-insular region; left MCA.	Mixed aphasia	6
Right frontal region; right temporal region; right fronto-parietal region; right insula and frontal lobes; right frontal subcortical region; parietal and occipital lobes; right occipital region; right MCA; right precentral gyrus.	Discursive processing deficits	7
	Pragmatics and lexical-semantic deficits	10
	No communication problems	6
	Discourse, pragmatics and lexical-semantic deficits.	2
Basal ganglia and insula; left MCA; left thalamus; area between the MCA and ACA; left thalamus and semioval centers; caudate nucleus; left frontal subcortical region; cortical-subcortical left prefrontal gyrus; left parietal; left periventricular region.	LHD, no aphasia	28

Caption: MCA = middle cerebral artery; ACA = anterior cerebral artery; LHD = left hemisphere damage.

### Instruments and procedures

Participants with LHD were assessed by the MTL-BR Battery^(4)^ in order to verify the presence of aphasia. Thus, the participants were divided into two groups: LHDna and LHDa. All participants completed the semantic and phonemic VF tasks from the MTL-BR^(4)^. The MTL-BR have satisfactory psychometric proprieties^(18,19)^. MTL-BR^(4)^ was designed to evaluate several components of oral and written language, including oral and written expression and comprehension, praxis abilities, and mathematical skills, in patients with a history of brain damage. The battery contains 20 subtests in addition to the two previously mentioned VF tasks^(4,18,19)^.

In the SVF task, the individual is asked to name as many animals as possible in 90 seconds. In the PVF task, they are asked to elicit the largest possible number of words starting with the letter “M” in a 90 second interval. Proper nouns are not counted as correct responses. The words elicited by participants in each 30-second block of the task are written down by the examiner. The tasks are also audio-recorded for fidelity purposes. This procedure also allows for the analysis of clustering and switching patterns.

To form a clustering it was necessary to include at least two consecutive words that belonged to the same semantic subcategory (eg. dog, cat) or first syllable (eg. monkey, moth) The switchings were analyzed when the participants alternate the subcategory to another, that is, the number of transitions between clustering ^(20)^.

In the present study, the number of words elicited in each 30 second interval as well as the instrument as a whole were analyzed for each type of VF task (SVF and PVF).

### Data analysis

Data were analyzed using SPSS, version 22, for Windows. Data distribution was assessed using Kolmogorov-Smirnov tests. Performance on the VF tasks (number of words elicited, number of clusters and switches) was compared between groups using one-way analysis of variance (ANOVA) followed by Bonferroni post-hoc tests. Group differences were considered significant at p < 05.

### Ethical procedures

The study was reviewed and approved by a university ethics committee under project number 04908/09. All participants provided written informed consent, as recommended by National Health Council Resolution 466/12.

## RESULTS

The performance of each participant group on the SVF task is shown in Table 2. In addition to the number of clusters and switches, the number of words elicited in each 30-second interval of the task, as well as the task as a whole, were compared between groups.

**Table 2:** Between-group comparisons of performance on the semantic verbal fluency task

	LHDa (*n* = 26)		RHD (*n* = 25)		LHDna (*n* = 28)		C (*n* = 25)		*F(3,100)*	*p*	*Post hoc*					
	*M*	*SD*	*M*	*SD*	*M*	*SD*	*M*	*SD*			LHDa X RHD	LHDa X LHDna	LHDa X C	RHD X LHDna	RHD X C	LHDna X C
Block 1	3.96	2.57	10.00	3.39	9.75	3.24	12.28	3.75	30.457	**≤ .001**	**≤ .001**	**≤ .001**	**≤ .001**	1.000	.090	**.035**
Block 2	.77	1.14	4.92	3.32	4.68	3.07	6.24	3.70	16.266	**≤ .001**	**≤ .001**	**≤ .001**	**≤ .001**	1.000	.712	.351
Block 3	.92	1.35	3.44	2.55	4.14	2.69	3.72	2.92	9.187	**≤ .001**	**.002**	**≤ .001**	**≤ .001**	1.000	1.000	1.000
Total	5.65	4.10	18.36	7.72	18.54	7.29	22.24	8.26	27.577	**≤ .001**	**≤ .001**	**≤ .001**	**≤ .001**	1.000	.320	.347
*Clusters*	1.23	1.03	4.16	1.89	4.18	1.66	5.52	2.62	24.041	**≤ .001**	**≤ .001**	**≤ .001**	**≤ .001**	1.000	.070	.064
*Switches*	.46	.76	3.12	1.86	3.18	1.66	4.52	2.62	22.130	**≤ .001**	**≤ .001**	**≤ .001**	**≤ .001**	1.000	**.049**	.054

**Caption:** LHDa = Left hemisphere damage, aphasia; RHD = Right hemisphere damage; LHDna = Left hemisphere damage, no aphasia; C = Control group; M= Mean; SD = Standard deviation.

The LHDa performed worse than all other participant groups on the SVF task as a whole. The RHD group made fewer switches than the control group, while the LHDna group elicited a smaller number of words than the control group in the first 30 second interval of the task (Table 2). The data pertaining to participant performance on the PVF task is shown in Table 3.

**Table 3:** Between-group comparisons of performance on the phonemic verbal fluency task

	LHDa (*n* = 26)		RHD (*n* = 25)		LHDna (*n* = 28)		C (*n* = 25)		*F(3,100)*	*p*	*Post hoc*					
	*M*	*SD*	*M*	*SD*	*M*	*SD*	*M*	*SD*			LHDa X RHD	LHDa X LHDna	LHDa X C	RHD X LHDna	RHD X C	LHDna X C
Block 1	1.38	1.58	6.24	2.67	5.79	2.85	7.64	2.80	29.402	**≤ .001**	**≤ .001**	**≤ .001**	**≤ .001**	1.000	.318	.054
Block 2	.50	1.11	3.40	2.40	2.79	2.10	5.16	2.38	22.399	**≤ .001**	**≤ .001**	**≤ .001**	**≤ .001**	1.000	**.019**	**≤ .001**
Block 3	.54	.81	3.56	2.45	2.68	2.20	3.68	2.10	13.704	**≤ .001**	**≤ .001**	**≤ .001**	**≤ .001**	.664	1.000	.423
Total	2.42	3.08	13.20	6.64	11.25	6.20	16.48	6.13	28.662	**≤ .001**	**≤ .001**	**≤ .001**	**≤ .001**	1.000	.264	**.007**
*Clusters*	.46	.71	3.04	1.97	2.46	1.82	4.04	2.41	17.461	**≤ .001**	**≤ .001**	**≤ .001**	**≤ .001**	1.000	.334	**.013**
*Switches*	.12	.33	2.08	1.91	1.39	1.69	3.04	2.41	12.629	**≤ .001**	**≤ .001**	.052	**≤ .001**	.940	.331	**.005**

**Caption:** LHDa = Left hemisphere damage, aphasia; RHD = Right hemisphere damage; LHDna = Left hemisphere damage, no aphasia; C = Control group; M= Mean; SD = Standard deviation.

The LHDa group performed worse than all other participant groups on every PVF score save for the number of switches, which did not differ between these individuals and the LHDna group. Significant differences were also observed between the RHD group and control participants, whereby the former elicited fewer words than the latter in the second block of the PVF task. The LHDna group also performed worse than control subjects on most PVF scores, save for the number of words elicited in the first and third blocks of the task.

The types of cluster formed by each participant group in the SVF and PVF tasks are described in Tables 4 and 5. In the first 30 seconds of the SVF task, the majority of participants, regardless of group, retrieved words from the farm animal, wild animal and domestic animal subcategories. These clusters continued to be the most frequent in later intervals of the task (Table 4), although the number of words elicited in these clusters tended to decrease. In the PVF task, most clusters were formed by words beginning with the same syllable (‘ma’, ‘me’, ‘mi’, ‘mo’, ‘mu’), although the semantic cluster “fruit” was also frequently used (Table 5).

**Table 4:** Classification of clusters produced in the semantic verbal fluency task by control participants and clinical subjects

Categories	Groups											
Time/block	LHDa			RHD			LHDna			Control		
0-30s	30-60s	60-90s	0-30s	30-60s	60-90s	0-30s	30-60s	60-90s	0-30s	30-60s	60-90s
Wild	4	2	1	18	9	10	20	16	12	23	12	13
Domestic	9	2	2	10	1	1	17	0	0	15	0	1
Farm	12	1	0	19	7	10	23	5	4	17	6	2
Sea	0	0	0	0	3	1	0	0	1	1	1	2
Insects	0	0	0	1	1	3	1	0	2	2	1	1
Birds	1	0	1	5	5	4	3	5	5	10	4	2
Reptiles	0	0	0	0	1	1	2	3	1	1	3	0
Phonological	1	0	0	0	3	0	2	1	0	0	1	1

**Caption:** LHDa = Left hemisphere damage, aphasia; RHD = Right hemisphere damage; LHDna = Left hemisphere damage, no aphasia; C = Control group.

**Table 5:** Classification of clusters produced in the phonemic verbal fluency task by control participants and clinical subjects

Categories	Groups											
Time/block	LHDa			RHD			LHDna			Control		
0-30s	30-60s	60-90s	0-30s	30-60s	60-90s	0-30s	30-60s	60-90s	0-30s	30-60s	60-90s
First syllable	2	1	1	26	11	12	28	15	9	28	14	18
Food	0	0	0	3	2	0	1	0	0	4	2	0
Objects	0	0	0	0	0	1	0	0	0	0	1	0
Fruit	3	0	0	8	0	0	6	0	1	6	1	1
Clothing	0	0	0	1	0	0	0	0	0	0	0	0
Semantic/other (master-mastery); (mechanics–mechatronics)	0	0	0	0	1	1	1	0	1	1	1	0

**Caption:** LHDa = Left hemisphere damage, aphasia; RHD = Right hemisphere damage; LHDna = Left hemisphere damage, no aphasia; C = Control group.

## DISCUSSION

As expected, the LHDa group obtained lower scores than all remaining subjects on the majority of FVS and PVF measures^(1,10,18,21,22)^. LHD is associated with particularly severe linguistic impairments, especially in patients with aphasia. This condition affects all areas of language to varying degrees, but is mainly associated with alterations in verbal expression and comprehension^(21,23)^. However, linguistic or communication skills are also known to be associated with the executive functions. Studies of patients with aphasia have shown that impairments in executive functions such as initiation, flexibility and planning also affect linguistic abilities^(23)^. Therefore, VF tasks may play an important role as an early screening measure for patients with aphasia, as they predict cognitive function as pertains to both language and executive processing.

RHD patients can present impairments related mainly to pragmatic, discursive, lexical-semantic and prosodic skills^(24)^. Studies shows that in verbal fluency tasks RHD patients have predominant recruitment of the left hemisphere or bilateral regions. The right hemisphere has a limited participation in language processing^(25)^.

Both control and clinical samples showed better performance in the first blocks of VF tasks, which is known to rely most heavily on automatic information processing^(15,26)^. This effect was especially evident in the first 15 seconds of the tasks. These results suggest that commonly used words may be easily available and automatically retrieved when the task begins. Once this set of words is exhausted, additional effort must be invested to retrieve further responses, which tend to be less numerous and more closely related to controlled recall, which relies more heavily on the executive functions^(27,28)^. However, the LHDna group performed worse than the control group in the first block of the SVF task, with the former producing significantly fewer words than the latter (Table 2). This may be due to between-group differences in the speed of initiation, since no additional differences were detected between LHDna subjects and controls on later blocks of the task.

Unlike the LHDna group, patients with RHD tend to respond to stimuli quickly and impulsively, increasing their error rates in neuropsychological tests^(29)^. Although the total number of words produced in the SVF task did not differ between the LHDna, RHD and control groups, the number of switches was significantly lower in the RHD group relative to control subjects. Unlike other participant groups, patients with RHD sought to exhaust every category after having selected it. This was observed in clusters such as domestic, wild or farm animals, the most commonly explored across all groups, as shown in Table 4. Switching depends primarily on skills such as cognitive flexibility, the search and use of retrieval strategies, and self-monitoring, which are often impaired in individuals with RHD^(8,29)^.

VF tasks usually require access to two types of lexical storage: long-term storage which can be quickly accessed and contains the majority of frequently used words; and a more extensive lexicon which is searched after the former is exhausted^(28)^. Concepts which are similar in meaning are stored together, increasing the speed of recall. SVF is thought to be easier than PVF, as it relies on the organization of semantic association networks^(28)^.

PVF, on the other hand, requires that words be retrieved based primarily on their lexical representation^(27)^. The suppression of the usual habit of searching for words based on meaning requires additional cognitive effort, and is closely associated with prefrontal cortical activity and the executive functions^(30)^.

In the present study, the lowest scores on the PVF task were obtained by the LHDa group. This may be attributable to the verbal nature of the task and the impairments displayed by patients with aphasia. The lack of differences in the number of switches between the LHDa and LHDna groups may be explained by the location of the lesions suffered by participants. Studies suggest that the contents of VF clusters are associated with specific features of each hemisphere^(8)^. Therefore, although cluster size did not differ between patients with right- versus left-hemisphere lesions, it appears that patients with LHD are less likely to switch between clusters when compared to patients with RHD and control subjects.

Like the LHDa group, patients in the LHDna group differed from the control group in terms of the number of clusters, switches, and words produced in the second block of the PVF task. According to the literature, the slower retrieval of words within clusters is associated with a reduction in the number of switches^(28)^. Additionally, studies of the relevance of the left cerebral hemisphere in the context of linguistic processing have demonstrated that damage to this region can cause major impairments in both semantic and phonological skills^(23)^.

The comparison between the RHD and control groups showed that the latter outperformed the former in block II of the PVF task. Our findings showed that patients with RHD produced a considerable number of words in block 1, but showed a decrease in performance in block 2, only to improve again in block 3. According to the literature, the last intervals of VF tasks rely more heavily on Executive Functions, since the words elicited at this point in the task are not as readily available in memory as those evoked in the beginning of the instrument^(27,28)^.

Verbal fluency tasks are cost-effective and quick to administer, which makes them ideal for use in several environments, including hospitals and outpatient clinics. Additionally, the quantitative and qualitative (clustering and switching) scores provided by these instruments can help detect language impairments and monitor patient progress, contributing to the diagnosis, follow-up and rehabilitation of patients with neurological damage. These observations underscore the applicability of verbal fluency tasks to patients with these conditions. The aim of this study was to compare the performance and strategies used by different clinical and control groups in verbal fluency tasks, highlighting the differences between these populations.

The present findings showed that participants with LHD, especially those with aphasia, experienced the greatest difficulties in both semantic and phonological fluency tasks, producing fewer clusters and making fewer switches relative to other clinical groups and control participants. Therefore, in addition to the language impairments commonly associated with aphasia, these individuals may have alterations in executive functions such as cognitive flexibility, which can also influence performance in this type of task. Furthermore, the results show that regardless of the injury site (left or right hemisphere) the word search strategies are quite similar for both FVS and FVF. Differing mainly only in the amount of words evoked.

Nevertheless, the present findings must be interpreted in light of some limitations, including a possible confounding effect of the time since stroke, which was not considered in the comparison between clinical groups. In this study the results are not statistically significant but they were very close to .05. Finally, it was not possible to identify the specific regions of the lesions in the LHDna and RHD cases, which did not allow comparing the data with the literature findings.
